# The effect of Huoxue Huayu decoction on restenosis after percutaneous coronary intervention in patients with coronary heart disease: A protocol for systematic review and meta-analysis

**DOI:** 10.1097/MD.0000000000028677

**Published:** 2022-01-28

**Authors:** Yi Hou, Xuhao Li, Xingxin Wang, Tiantian Dong, Jiguo Yang

**Affiliations:** College of Acupuncture and Massage, Shandong University of Traditional Chinese Medicine, Jinan, Shandong Province, China.

**Keywords:** coronary heart disease, coronary restenosis, Huoxue Huayu decoction, meta-analysis, percutaneous coronary intervention, protocol

## Abstract

**Background:**

Percutaneous coronary intervention (PCI), as the most common treatment for coronary heart disease (CHD), has the advantages of simple operation, minimal invasion, rapid reconstruction, and vessels opening. The problem, however, is that many patients develop restenosis within 6 months after PCI. In traditional Chinese medicine (TCM), Huoxue Huayu decoction (HXHYD) is widely used to treat cardiovascular diseases, and its important role as a complementary and alternative therapy for the prevention and treatment of post-PCI restenosis in CHD patients has been extensively reported. However, controversy exists among different studies. Therefore, we collected relevant randomized controlled trials for a meta-analysis to assess the efficacy and safety of HXHYD in the prevention of post-PCI restenosis in patients with CHD.

**Methods:**

Randomized controlled trials of HXHYD in the prevention of post-PCI restenosis in patients with CHD will be retrieved from PubMed, Embase, Web of Science, Cochrane Library, China National Knowledge Infrastructure, Wan Fang Database, Chinese Biomedical Literature Database, VIP Database for Chinese Technical Periodicals, and Clinical Trial Register. The 2 authors will independently conduct the literature search, literature screening, data extraction, and quality assessment. Data analysis will be performed using STATA 14.0.

**Results:**

The results of this meta-analysis will be submitted to a peer-reviewed journal for publication.

**Conclusion:**

This study will provide high-quality, evidence-based medical evidence for the efficacy and safety of HXHYD in the prevention of post-PCI restenosis in patients with CHD.

**Ethics and dissemination:**

Ethical approval is not required for this study. The systematic review will be published in a peer-reviewed journal, presented at conferences, and shared on social media platforms. This review would be disseminated in a peer-reviewed journal or conference presentations.

**OSF REGISTRATION NUMBER:**

DOI 10.17605/OSF.IO/PNZSM.

## 1 Introduction

Coronary atherosclerotic heart disease refers to a heart disease caused by myocardial ischemia, hypoxia, or necrosis due to lumen stenosis or occlusion caused by atherosclerosis of the coronary arteries, referred to as coronary heart disease (CHD).^[1,2]^ The current treatment for CHD focuses on drug therapy, coronary artery bypass grafting, and percutaneous coronary intervention (PCI). PCI is the main treatment for CHD.^[3,4]^ However, post-PCI restenosis remains a challenging clinical problem.^[5,6]^ Restenosis is defined as a lumen occlusion of more than 50%. This usually occurs within 6 months after PCI, and the mechanism for this is not fully understood.

With the continuous update and development of drug-eluting stents and new anticoagulant drugs, the incidence of restenosis has decreased significantly, but restenosis still occurs in 10% of patients.^[7]^ The conventional drug therapy in Western medicine is not effective in patients with post-PCI restenosis, which makes the search for drugs to prevent post-PCI restenosis an urgent problem.

In China, traditional Chinese medicine (TCM) is widely used for the prevention and treatment of cardiovascular diseases.^[8,9]^ Clinical studies have found that TCM can reduce the incidence of post-PCI restenosis.^[10–14]^ CHD falls into the category of blood stasis evidence with blood stasis and phlegm obstruction, Qi deficiency and cold clotting in TCM.^[15]^ Post-PCI restenosis is caused by damage to the arteries and channels based on Qi deficiency, internal stasis of blood and paralysis of the heart channels.^[16]^ Therefore, tonifying Qi and activating blood stasis is the general principle of treatment for patients with post-PCI restenosis.^[17]^

Huoxue Huayu decoction (HXHYD) is composed of Radix Angelicae Sinensis, Rhizoma Chuanxiong, Radix Paeoniae Alba, and Peach kernel etc, which can reduce myocardial reperfusion injury by activating blood circulation and resolving blood stasis, regulating Qi and relieving pain.^[18]^ In recent years, several studies have shown the importance of HXHYD as a complementary and alternative therapy to prevent post-PCI restenosis in patients with CHD.^[17,19,20]^ However, controversy exists among different studies. Therefore, we collected relevant randomized controlled trials (RCTs) for a meta-analysis to assess the efficacy and safety of HXHYD for the prevention of post-PCI restenosis in patients with CHD.

## 2 Methods

### 2.1 Study registration

The protocol of the systematic review has been registered on Open Science Framework (registration number: DOI 10.17605/OSF.IO/PNZSM). It was reported by following the guideline of Preferred Reporting Items for Systematic Reviews and Meta-analysis Protocol statement.^[21]^

### 2.2 Inclusion criteria for study selection

#### 2.2.1 Type of studies

RCTs of HXHYD for preventing post-PCI restenosis in patients with CHD will be included.

#### 2.2.2 Type of participants

All patients with CHD after PCI were included.

#### 2.2.3 Types of intervention

The control group was given Western medicine alone. The intervention group was treated with HXHYD in combination with Western medicine.

#### 2.2.4 Outcome measurements

Primary outcomes: coronary restenosis was recorded as the primary outcome, defined as stenosis >50% of the diameter.

Secondary outcomes: major adverse cardiac events and the Seattle Angina Questionnaires.

### 2.3 Exclusion criteria

1. Duplicate publications in the literature.
2. Case series, case reports, reviews, etc.
3. Animal experiments and cellular experiments.

### 2.4 Data sources and search strategy

This study conducted a literature search in PubMed, Embase, Web of Science, Cochrane Library, China National Knowledge Infrastructure, Wan Fang Database, Chinese Biomedical Literature Database, VIP Database for Chinese Technical Periodicals, and Clinical Trial Register. We made a final search in January 2022. The search strategy for Pubmed is displayed in Table 1. Similar search strategies will be applied to other electronic databases.

**Table 1:** PubMed search strategy.

Number	Search terms
#1	Coronary Disease[MeSH]
#2	Coronary Heart Disease[Title/Abstract]
#3	Coronary Diseases[Title/Abstract]
#4	Coronary Heart Diseases[Title/Abstract]
#5	Disease, Coronary[Title/Abstract]
#6	Disease, Coronary Heart[Title/Abstract]
#7	Diseases, Coronary[Title/Abstract]
#8	Diseases, Coronary Heart[Title/Abstract]
#9	Heart Disease, Coronary[Title/Abstract]
#10	Heart Diseases, Coronary[Title/Abstract]
#11	or/1-10
#12	Restenosisin-stent[Title/Abstract]
#13	In-stent restenosis[Title/Abstract]
#14	ISR[Title/Abstract]
#15	Coronary Restenosis[MeSH]
#16	Coronary Restenoses[Title/Abstract]
#17	Restenoses, Coronary[Title/Abstract]
#18	Restenosis, Coronary[Title/Abstract]
#19	Restenosis [Title/Abstract]
#20	or/12-19
#21	Huoxue Huayu Decoction[Title/Abstract]
#22	Huoxue Huayu Tang[Title/Abstract]
#24	or/21-22
#24	Randomized Controlled Trials as Topic[MeSH]
#25	Clinical Trials, Randomized[Title/Abstract]
#26	Controlled Clinical Trials, Randomized[Title/Abstract]
#27	Trials, Randomized Clinical[Title/Abstract]
#28	Random∗[Title/Abstract]
#29	or/24-28
#30	#11 and #20 and #24 and #29

### 2.5 Data collection and analysis

#### 2.5.1 Study selection

**Figure 1:**
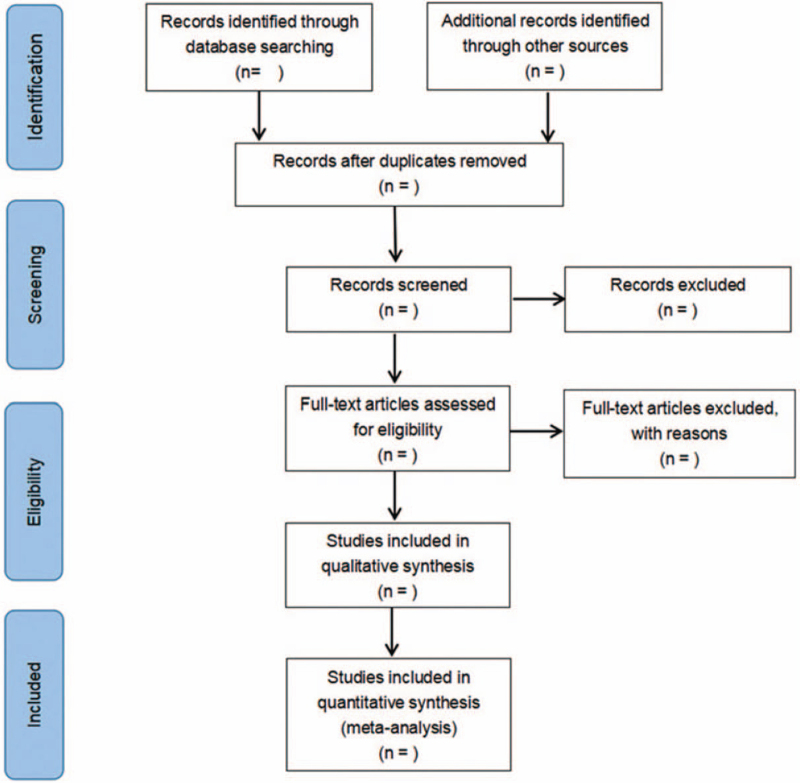
Flow diagram of literature retrieval.

The 2 researchers performed literature screening and data extraction independently. First, the retrieved literature will be imported into the literature management software Endnote for duplicate data removal. After reading the title, abstract and keywords, these 2 researchers will independently select relevant studies. After reading the full text, these 2 researchers will screen the rest of the literature. Any discrepancies between the 2 reviewers will be discussed and resolved by a third researcher. If the required information is missing, the authors will be contacted. The screening flow chart of this study is demonstrated in Figure 1.

#### 2.5.2 Data extraction

The 2 researchers independently extracted data using a pre-determined data extraction form. Contents included general information (title, authors, date of publication, literature source), participants (number of cases, age, sex, duration of disease), interventions (dose of HXHYD, duration and frequency of intervention, duration of follow-up), outcome indicators (coronary restenosis, major adverse cardiac events, and the Seattle Angina Questionnaires) and literature quality evaluation details.

#### 2.5.3 Dealing with missing data

If the data of the required study are incomplete or not reported in the study, the researcher will contact the first author or other authors of the study by phone or email. If the required data are not available, we will conduct a descriptive analysis, instead of meta-analysis, and exclude these studies if necessary.

### 2.6 Quality assessment

RCT quality was rated according to the risk of bias tool from the Cochrane Collaboration. The 2 investigators independently and blindly checked the studies. Any discrepancies between the 2 investigators were resolved by consensus.

### 2.7 Statistical analysis

All of the above statistical analyses were performed with Stata 14.0 (StataCorp LLC, college station, TX). Relative risk with 95% confidence interval will be used to measure the curative effect for dichotomous variables, and the standardized mean difference with 95% confidence interval will be used for continuous data. The *I*^2^ statistic and Cochran *Q* test were used to assess homogeneity among the RCTs. If *I*^2^ is <50%, a fixed-effects model is applied. If *I*^2^ is >50%, a random-effects model is applied. If *I*^2^ is >85%, no meta-analyses is performed.

### 2.8 Subgroup analysis

Subgroup analyses were conducted based on dose of HXHYD and sample size.

### 2.9 Sensitivity analysis

In order to test the stability of the meta-analysis results, sensitivity analysis was performed by removing each study in turn to verify the consistency of the results.

### 2.10 Reporting bias

Funnel plots were used to evaluate publication bias.

### 2.11 Ethics and dissemination

Since the program does not include the recruitment of patients and the collection of personal information, the approval of the Ethics Committee is not required.

## 3 Discussion

CHD is the most common cardiovascular disease, primarily due to coronary artery stenosis or obstruction caused by atherosclerosis.^[22]^ PCI is one of the important tools for the treatment of CHD, but the consequent occurrence of restenosis severely affects the efficacy of PCI in CHD patients.^[23–25]^ Although drug-eluting stents can reduce the incidence of restenosis in the short term, the incidence of restenosis remains high in the end. Therefore, it is particularly important to find a safe and effective treatment to prevent post-PCI restenosis in CHD patients. In recent years, a number of studies have demonstrated the efficacy of HXHYD in preventing and treating post-PCI restenosis in patients with CHD. However, the efficacy of different studies is still controversial. In this study, for the first time, a meta-analysis was used to evaluate the efficacy and safety of HXHYD for the prevention and treatment of post-PCI restenosis in patients with CHD. Therefore, this study is expected to provide a reliable basis for the clinical application of HXHYD to prevent post-PCI restenosis in patients with CHD.

## Author contributions

**Conceptualization:** Yi Hou, Jiguo Yang.

**Data curation:** Yi Hou, Xuhao Li.

**Formal analysis:** Xuhao Li.

**Funding acquisition:** Jiguo Yang.

**Investigation:** Xuhao Li, Tiantian Dong.

**Methodology:** Xuhao Li, Tiantian Dong.

**Project administration:** Jiguo Yang.

**Resources:** Xingxin Wang, Tiantian Dong, Jiguo Yang

**Software:** Xingxin Wang, Tiantian Dong

**Supervision:** Jiguo Yang.

**Validation:** Xingxin Wang.

**Visualization:** Xingxin Wang.

**Writing – original draft:** Yi Hou, Jiguo Yang.

**Writing – review & editing:** Yi Hou, Jiguo Yang.
